# Deep Brain Stimulation of the Subthalamic Nucleus in Patients with Parkinson Disease with Prior Pallidotomy or Thalamotomy

**DOI:** 10.3390/brainsci8040066

**Published:** 2018-04-16

**Authors:** Elena A. Khabarova, Natalia P. Denisova, Aleksandr B. Dmitriev, Konstantin V. Slavin, Leo Verhagen Metman

**Affiliations:** 1 Functional Neurosurgery Department, “Federal Neurosurgical Center”, Novosibirsk 630087, Russia; n_denisova@neuronsk.ru (N.P.D.); a_dmitriev@neuronsk.ru (A.B.D.); 2 Department of Neurosurgery, University of Illinois, Chicago, IL 60612, USA; kslavin@uic.edu; 3 Department of Neurological Sciences, Rush University, Chicago, IL 60612, USA; lverhage@rush.edu

**Keywords:** Parkinson disease, STN DBS, pallidotomy, thalamotomy

## Abstract

**Objective.** To evaluate the efficacy of deep brain stimulation of the subthalamic nucleus (STN DBS) in patients with Parkinson disease (PD) who previously underwent lesioning of the basal ganglia. **Material and methods.** The study included 22 patients who underwent STN DBS. Eleven patients had undergone prior unilateral pallidotomy (*n* = 6) or VL/VIM thalamotomy (*n* = 5) while the other 11 patients had not. The primary outcome was the change from baseline in the motor subscore of the Unified Parkinson Disease Rating Scale (UPDRS-III) 12 months after STN DBS. Secondary outcomes included change in motor response complications (UPDRS-IV) and change in levodopa equivalent daily dose (LEDD). **Results.** In the group with prior lesioning UPDRS-III improved by 45%, from 51.5 ± 9.0% (range, 35–65) to 26.5 ± 8.4 (range, 21–50) (*p* < 0.01) and UPDRS-IV by 75%, from 8.0 ± 2.01 (range, 5–11) to 2.1 ± 0.74 (range, 1–3) (*p* < 0.01). In the group without prior lesioning UPDRS-III improved by 61%, from 74.2% ± 7.32 (range, 63–82) to 29.3 ± 5.99 (range, 20–42) (*p* < 0.01) and UPDRS-IV by 77%, from 9.1 ± 2.46 (range, 5–12) to 2.0 ± 1.1 (range, 1–4) (*p* < 0.01). Comparing the two groups (with and without lesioning) no significant differences were found either in UPDRS-III (*p* > 0.05) or UPDRS-IV scores (*p* > 0.05) at 12 months post-DBS. The LEDD was reduced by 51.4%, from 1008.2 ± 346.4 to 490.0 ± 194.3 in those with prior surgery (*p* < 0.01) and by 55.0%, from 963.4 ± 96.2 to 433.3 ± 160.2 in those without (*p* < 0.01).UPDRS-III improved by 51.8%, from 53.7 ± 4.6 (range, 50–62) to 25.0 ± 3.8 (range, 21–31) in those with prior pallidotomy (*p* < 0.01), and by 37.5%, from 48.8 ± 12.6 (range, 35–65) to 29.8 ± 13.6 (range, 22–50) in those with prior thalamotomy (*p* < 0.01). This numerical difference in improvement was not statistically significant (*p* > 0.05). **Conclusion.** Our comparative study indicates that bilateral STN DBS is effective and can be used in patients with Parkinson disease with prior unilateral stereotactic destructive operations on subcortical structures. The results in our patient cohort are generally consistent with previously published reports of smaller series from multiple centers worldwide.

## 1. Introduction

Idiopathic Parkinson disease (PD) is the second most common neurodegenerative disease (after Alzheimer disease) [1]. The prevalence of PD among individuals older than 65 years old is nearly 1%. Disease onset is most commonly between age 60 and 70, but in 15% of patients the disease starts before age 45 [1]. Although dopaminergic therapy is effective for the motor symptoms of PD, after 5 years of levodopa therapy more than 50% of patients develop motor response fluctuations while the risk of drug-induced dyskinesias increases every year by 10% [2]. Once motor response complications start to interfere with patients’ activities of daily living or quality of life surgical treatment strategies become an option. While deep brain stimulation is the preferred treatment in many countries, lesioning procedures such as pallidotomy and thalamotomy are still relevant, especially in areas where DBS is not available or not covered by health insurance. Unilateral pallidotomy and thalamotomy effectively reduce contralateral parkinsonian symptoms but bilateral lesional procedures have been reported to be associated with significant side effects. Investigators have reported a 30–60% incidence of speech and cognitive disorders after bilateral ventrolateral (VL) thalamotomy [3] and a 17% incidence of speech and cognitive disorders after bilateral pallidotomy [3,4]. It therefore makes sense to offer patients who already have a unilateral lesion, not a contralateral destructive procedure, but rather a non-destructive DBS procedure. STN DBS is known to improve all cardinal symptoms of PD, including tremor, bradykinesia and rigidity and also improve quality of life of patients with advanced as well as moderate PD [5]. The effectiveness of STN DBS after destructive interventions is insufficiently studied. In this study, we report our experience with STN DBS in PD patients who had prior lesional procedures and compare their outcomes with those of PD patients who did not have previous destructive surgery.

## 2. Materials and Methods

Twenty-two patients who underwent bilateral STN DBS at the Federal Neurosurgical Center in Novosibirsk between 2013 and 2016 participated in this study (Table 1). Eleven consecutive patients (7 men, 4 women) had already undergone unilateral destructive surgery in the past (6 pallidotomy, 5 thalamotomy) but lack of ipsilateral control of motor symptoms and progression of the disease on the contralateral side prompted additional surgery. In this “lesioned group” the mean age at the time of the lesioning procedure was 54.7 ± 5.5 years (range: 47–64) and at the time of STN DBS 55.9 ± 5.3 years (range, 47–65). The mean disease duration was 12.7 ± 2.1 years (range: 5–20). The time between surgeries was 14.3 ± 5.4 months (range: 5–26). The remaining 11 patients (7 men, 4 women) also underwent bilateral STN DBS but did not have prior lesional surgery. In this “unlesioned group” the mean age was 54.8 ± 4.3 years (range, 48–61) and the disease duration 12.4 ± 3.1 years (range, 7–16). We used the same indications and selection criteria for STN DBS as those published in the literature in the past [6]; these criteria were identical for both groups.

Surgical intervention was performed using a standard protocol: the electrodes were implanted using a frame-based stereotactic procedure (CRW stereotactic frame, StereoCalc and ImageFusion software, Integra, Allmendstrasse, Wohlen) and intraoperative microelectrode recording (NeuroNav, Alpha-Omega Engineering, Nazareth Illit, Israel) under local anesthesia. The same frame-based approach and local anesthesia were used for lesioning prior to the DBS procedure. The devices used for DBS were conventional (omnidirectional) 4-contact electrodes and 8-contact generators from Medtronic (*n* = 22) and St. Jude Medical (*n* = 2).

The minimal follow up period in patients with STN DBS was 12 months, the maximal 30 months (mean: 21.4 ± 5.2 months). The primary outcome was the change from baseline in the motor subscore of the Unified Parkinson Disease Rating Scale (UPDRS-III) 12 months after STN DBS. Secondary outcomes included change in motor response complications (UPDRS-IV) and change in levodopa equivalent daily dose (LEDD). LEDD was calculated using the following formula: *x* = [(levodopa + levodopa CR * 0.7) * 1.1, if on COMT inhibitor] + pramipexole * 100 + ropinirole * 33 [7]. Programming of the neurostimulator was performed 1 month after surgery. Stimulation parameters were selected based on the best clinical effect on the motor symptoms with the least possible side effects (dysarthria, levodopa-induced dyskinesias).

None of the patients suffered significant complications because of the surgery.

### Statistics

Data analysis was performed with SPSS 22.0 statistics software package. For all variables expected values and standard deviations were calculated. Comparison of changes in the pre- and postsurgical UPDRS scores (Part III and IV), medication requirements (LEDD), and dyskinesia scores between two groups was performed using the Mann-Whitney U-test. Statistical significance was assumed at *p* < 0.05.

## 3. Results

### 3.1. Unilateral Pallidotomy or Thalamotomy

Six patients underwent a right sided lesion and the remaining 5 a left sided lesion. Six months after lesioning UPDRS-III-OFF the scores in this group improved by 17%, from 57.2 ± 4.9 (range, 52–67) to 47.5 ± 9.6 (range, 33–65) (*p* < 0.01) (Table 2).

### 3.2. STN DBS

Prior to STN DBS, UPDRS-III-OFF scores were higher in the unlesioned group (74.2 ± 7.32 (63–82) than in the lesioned group (51.5 ± 9.0 (35–65) (*p* < 0.05). This is likely due to persistent benefit from the destructive procedure and plays a role in the differences between the absolute and percentage improvement between the two groups. In the unlesioned group UPDRS-III-OFF scores improved by 61%, from 74.2 ± 7.32 (range, 63–82) to 29.3 ± 5.99 (range, 20–42) (*p* < 0.01) and UPDRS-IV by 77%, from 9.1 ± 2.46 (range, 5–12) to 2.0 ± 1.1 (range, 1–4) (*p* < 0.01). On the other hand, in the lesioned group UPDRS-III-OFF scores 12 months post-DBS improved by 45%, from 51.5 ± 9.0 (range, 35–65) to 26.5 ± 8.4 (range, 21–50) (*p* < 0.01) and UPDRS-IV by 75%, from 8.0 ± 2.01 (range, 5–11) to 2.1 ± 0.74 (range, 1–3) (*p* < 0.01). Importantly, comparison of the two groups at the 12-month time-point revealed no differences either in UPDRS-III-OFF scores (26.5 ± 8.4 for the lesioned group, versus 29.3 ± 5.99 for the unlesioned group; *p* > 0.05) or UPDRS-IV scores (2.1 ± 0.74 for the lesioned group, versus 2.0 ± 1.1; *p* > 0.05) (Table 1). This likely represents a floor effect of UPDRS-III OFF scores in patients with advanced PD.

Patients of the lesioned group had a numerically different degree of improvement with stimulation depending on the lesion target (see Table 3). In those with prior pallidotomy UPDRS-III-OFF scores improved by 51.8%, from 53.7 ± 4.6 (range, 50–62) to 25.0 ± 3.8 (range, 21–31) (*p* < 0.01), whereas in those with prior thalamotomy UPDRS-III-OFF score decreased by 37.5%, from 48.8 ± 12.6 (range, 35–65) to 29.8 ± 13.6 (range, 22–50) (*p* < 0.01). This numerical difference in improvement between targets was not statistically significant (*p* > 0.05).

The LEDD was reduced by 51.4%, from 1008.2 ± 346.4 to 490.0 ± 194.3 (*p* < 0.01) in those with prior surgery and by 55.0% from 963.4 ± 96.2 to 433.3 ± 160.2 (*p* < 0.01). There was no difference in the degree of LEDD reduction between the 2 groups (*p* > 0.05).

## 4. Discussion

Stereotactic lesioning using radiofrequency thermodestruction is a time-tested and established modality for reliable control of motor PD symptoms that is used during advanced stages of the disease [8]. For medically refractory PD tremor the procedure of choice in the past has been the ventro-lateral (VL)/ventral-intermedius (VIM) thalamotomy [9,10,11]. Unilateral pallidotomy (destruction of the internal segment of the globus pallidus, GPi) improves all cardinal symptoms of PD. It is somewhat less effective (compared to thalamotomy) in controlling PD tremor but substantially reduces drug-induced dyskinesias in contralateral limbs (with lesser effect on ipsilateral dyskinesias) and ameliorates motor fluctuations [9]. Although unilateral lesioning has been determined to be both safe and effective, both the frequency and severity of intraoperative and postoperative complications appear to increase significantly with bilateral lesioning [3].

Moreover, despite the excellent initial clinical effect encountered when the lesion is performed in the optimal location, it is estimated that 10–15% of patients develop recurrence of their preoperative symptoms within 1–2 years after the surgery, particularly in the ipsilateral limbs and axial musculature, most likely due to disease progression [10]. This may translate into a need for repeated surgical intervention.

To avoid complications associated with bilateral destructive interventions, Siegfried and Lippitz [12] in 1982 started using chronic stimulation of subcortical structures in patients with PD previously subjected to stereotactic lesioning. The current era of DBS for movement disorders started with the seminal report on combined VIM thalamotomy and stimulation for bilateral PD by Benabid et al. [13], whose subsequent pioneering approach of STN DBS dramatically changed the neurosurgical management of PD [14]. Since then, DBS has become the intervention of choice in the treatment of PD [15].

Among the main benefits of DBS are the minimal brain tissue injury associated with electrode implantation and the reversibility of neurostimulation effects. Modern neurostimulators make it possible to noninvasively adjust the stimulation program using multiple parameters (i.e., monopolar or bipolar stimulation mode, electrode polarity, frequency, pulse width and amplitude of stimulation) [16]. This allows the clinician to find an individually tailored effective and comfortable treatment program for each patient and to minimize stimulation related side effects [17].

Autopsy data of DBS patients who died of unrelated causes indicate that surgical intervention and long-term electrical exposure do not cause noticeable damage to structures around the active electrode [18]. In another study, Meissner and colleagues found no evidence of tissue damage from electrical stimulation [19] when approved hardware was used at clinically relevant parameters. The limited invasiveness of the DBS procedure reduces the incidence of persistent neurologic complications that may occur with destructive interventions [20,21]. Taken together, it is now considered safe to perform simultaneous bilateral surgery while keeping complication rates low [4,10,11].

There are few data in the literature on the effectiveness of STN DBS in patients with previous destructive interventions. A study by Kleiner-Fisman et al. in 2004 [22] showed that previous unilateral pallidotomy does not worsen the effectiveness of subsequent bilateral STN DBS. In addition, the intraoperative electrophysiological findings in their study were no different between the control group of 25 patients without previous lesioning and the group of 8 patients with previous pallidotomy.

Our results suggest that in the group of bilateral STN DBS in patients with previous unilateral pallidotomy and thalamotomy the degree of improvement in UPDRS-III OFF scores is lower than in the unlesioned group. This is to a large degree due to lower UPDRS-III-OFF scores in the lesioned group at baseline, likely due to persistent/residual benefit from the earlier unilateral stereotactic destruction. The UPDRS-III-OFF scores at the 12-month time point were very similar and seem to be appropriate for patients with advanced disease. In this regard, our findings are similar to those reported by Ondo et al. in 2006 [7] who analyzed 10 patients with previous pallidotomy and STN DBS and compared them with age-matched controls without previous lesioning. Moreover, our study indicates that motor symptoms after STN DBS in the group with previous thalamotomy improved to a lesser degree compared to those with prior pallidotomy.

In terms of severity of dyskinesias, the degree of improvement was similar in both previously lesioned and non-lesioned groups, which is in line with previous studies on this subject [7,22]. The reduction in LEDD 12 months after STN DBS was also not different between two groups.

In the past, a concern was raised [7] that previous thalamotomy could result in less accurate STN electrode placement on the side of prior destruction. Also, Zaidel et al. in 2008 [23] reported reduction of the neuronal activity in STN after previous pallidotomy based on experience with 6 patients (one of them with previous bilateral pallidotomy). We did not specifically analyze the intraoperative microelectrode recording data, but our limited experience indicates no particular issues in either anatomical (radiographic) or physiological STN localization for DBS purposes.

Currently, there is a general reluctance to consider STN DBS in patients with previous destructive lesions. As a matter of fact, the current Medicare rules specifically mention that “DBS is not reasonable and necessary and is not covered for essential tremor or Parkinson disease patients with previous movement disorder surgery within the affected basal ganglion” [24]. Our experience, as well as the earlier series from Toronto, Houston, and Jerusalem [7,22,23], clearly indicates that previous destructive lesioning does not negatively affect the clinical safety and effectiveness of bilateral STN DBS and that in those patients with prior destructive interventions, STN DBS should be strongly considered in cases of symptomatic recurrence and disease progression.

## 5. Study Limitations

The main limitations of this study are its small size, observational nature, and non-randomized approach. It is hard to conceive a prospective study that would randomize patients toward the treatment paradigm that is rarely if ever used in most clinical centers. The introduction of different means of stereotactic lesioning (such as radiosurgery and focused ultrasound) may necessitate prospective randomized investigations [25,26], but it is doubtful that DBS will be compared to a combination of lesioning and DBS to establish efficacy and/or safety of sequential use of two modalities.

Since this was an observational study in a small convenience sample, no power analysis was performed. As a consequence, the possibility of a type 2 error should be recognized and weakens the confidence that there was no true difference between the two groups. However, the difference between UPDRS-III-OFF scores 12 months post-DBS was less than 3 points, and in favor of the lesioned group. Furthermore, the effect of STN DBS on dyskinesia severity and LEDD reduction were very similar as well, supporting our finding that STN DBS improves motor symptoms to the same level in either group.

In terms of data analysis, the lack of appendicular UPDRS scores for each side limits our ability to determine whether bilateral DBS simply improved motor symptoms by stimulating the hemisphere contralateral to the prior lesion or also by potentiating the beneficial lesioning effects on the ipsilateral hemisphere.

## 6. Conclusions

Our comparative study indicates that bilateral DBS STN is effective and can be used in patients with Parkinson disease who had previously undergone unilateral stereotactic destructive operations on subcortical structures. The results in our patient cohort are generally consistent with previously published reports of smaller series from multiple centers worldwide.

## Figures and Tables

**Table 1:** Characteristics of patients in the lesioned group and unlesioned group.

Patient Characteristics	Lesioned Group (*n* = 11)	Unlesioned Group (*n* = 11)
Age at the time of STN DBS	55.9 ± 5.3 (47–65)	54.8 ± 4.3 (48–61)
Sex	7 m (56%)	7 m (56%)
UPDRS-III-OFF score pre-STN DBS	51.5 ± 9.0 * (35–65)	74.2 ± 7.32 * (63–82)
UPDRS-III-OFF score 12 months post-STN DBS	26.5 ± 8.4 (21–50) 45%	29.3 ± 5.99 (20–42) 61%
UPDRS-IV pre-STN DBS	8.0 ± 2.01 (5–11)	9.1 ± 2.46 (5–12)
UPDRS-IV-score 12 months post-STN DBS	2.1 ± 0.74 (1–3) 75%	2.0 ± 1.1 (1–4) 77%

* *p* ≤ 0.05; values are presented as mean (range).

**Table 2:** Characteristics of the patients in the lesioned group (*n* = 11). Please reformat so the last row of table fits on one line.

Previous Lesion						Subsequent of STN DBS			
Sex	Age at Time of Lesion (Years)	Target	Hemisphere	UPDRS-III-OFF Score Pre-Lesion	UPDRS-III-OFF Score 6 Months Post-Lesion	Age at Time of DBS (Years)	Hemisphere	UPDRS-III-OFF Score Pre-DBS	UPDRS-III-OFF Score 12 Months Post-DBS
m	54	Gpi	R	55	45	55	B	55	25
f	62	Vim	L	59	54	63	B	54	25
m	53	Gpi	L	67	62	53	B	62	31
m	47	Vim	R	65	65	47	B	65	50
m	59	Vim	R	57	33	60	B	35	22
f	64	Gpi	L	52	40	65	B	50	28
f	47	Gpi	R	57	46	50	B	54	22
m	51	Vim	R	56	39	53	B	37	22
f	57	Gpi	R	53	49	57	B	50	23
m	53	Gpi	L	56	46	55	B	51	21
m	55	Vim	L	52	44	57	B	53	22
	54.7 ± 5.5 (47–64)			57.2 ± 4.9 (52–67)	47.5 ± 9.6 (33–65)	55.9 ± 5.3 (47–65)		51.5 ± 9.0 (35–65)	26.5 ± 8.4 (21–50)

UPDRS—Unified Parkinson Disease Rating Scale; R—right hemisphere; L—left hemisphere; B—bilateral implantation; values are presented as mean (range); m—male; f—female.

**Table 3:** The lesioned group by target.

GPI					VIM				
Sex	Age at the Time of Lesion (Years)	Disease Duration (Years)	UPDRS-III-OFF Score Pre-DBS	UPDRS-III-OFF Score 12 Months Post-STN DBS	Sex	Age at the Time of Lesion (Years)	Disease Duration (Years)	UPDRS-III-OFF Score Pre-DBS	UPDRS-III-OFF Score 12 Months Post-STN DBS
m	55	15	55	25	f	63	11	54	25
m	53	13	62	31	m	47	5	65	50
f	65	12	50	28	m	60	11	35	22
f	50	15	54	22	m	53	20	37	22
f	57	10	50	23	m	55	12	53	22
m	53	11	51	21					
3м/3f	55.5 ± 5.2 (50–65)	12.7 ± 2.1 (10–15)	53.7 ± 4.6 (50–62)	25.0 ± 3.8 (22–31)	4м/1f	55.6 ± 6.2 (47–63)	11.8 ± 5.4 (5–20)	48.8 ± 12.6 (35–65)	29.8 ± 13.6 (22–50)
			51.8% *					37.5% *	

* *p* > 0.05; values are presented as mean (range); m—male; f—female.
